# Transvaginal perfusion of G-CSF for infertile women with thin endometrium in frozen ET program: A non-randomized clinical trial

**Published:** 2014-10

**Authors:** Maryam Eftekhar, Mozhgan Sayadi, Farideh Arabjahvani

**Affiliations:** *Research and Clinical Center for Infertility, Shahid Sadoughi University of Medical Sciences, Yazd, Iran.*

**Keywords:** *Thin endometrium*, *Granulocytecolonystimulatingfactor*, *Frozenthawedembryotransfer*, *Pregnancyrate*

## Abstract

**Background:** We often see patients with a thin endometrium in ART cycles, in spite of standard and adjuvant treatments. Improving endometrial growth in patients with a thin endometrium is very difficult. Without adequate endometrial thickness these patients, likely, would not have reached embryo transfer.

**Objective:** We planned this study to investigate the efficacy of intrauterine granulocyte colony-stimulating factor (G-CSF) perfusion in improving endometrium, and possibly pregnancy rates in frozen-thawed embryo transfer cycles.

**Materials and Methods: **This is a non-randomized intervention clinical trial. Among 68 infertile patients with thin endometrium (-7 mm) at the 12^th^-13^th^ cycle day, 34 patients received G-CSF. G-CSF (300 microgram/1mL) to improve endometrial thickness was direct administered by slow intrauterine infusion using IUI catheter. If the endometrium had not reached at least a 7-mm within 48-72 h, a second infusion was given. Endometrial thickness was assessed by serial vaginal ultrasound at the most expanded area of the endometrial stripe.

**Results: **The cycle was cancelled in the patients with thin endometrium (endometrial thickness below 7mm) until 19^th^ cycle day ultimately The cycle cancelation rate owing to thin endometrium was similar in G-CSF group (15.20%), followed by (15.20%) in the control group (p=1.00). The endometrial growth was not different within 2 groups, an improvement was shown between controlled and G-CSF cotreated groups, with chemical (39.30% vs. 14.30%) and clinical pregnancy rates (32.10% vs. 12.00%) although were not significant.

**Conclusion:** Our study fails to demonstrate that G-CSF has the potential to improve endometrial thickness but has the potential to improve chemical and clinical pregnancy rate of the infertile women with thin endometrium in frozen-thawed embryo transfer cycle.

## Introduction

The endometrium has received much less attention in reproductive research compared with the ovary, fertilization, or embryo development (1).(Casper 2011) However, the endometrium is essential for embryo implantation. Exact and specific endometrial maturational development is crucial in allowing implantation following assisted reproduction (2). Endometrial thickness has long been used as a marker of receptivity of the endometrium and as a prognostic factor in embryo transfers (3). An adequate thickness of endometrium is critical for a successful pregnancy (4). Endometrium below 7 mm in thickness is widely considered sub-optimal for transfer and associated with reduced pregnancy chances. Therefore, thin endometrium is an extremely poor factor that interferes with an ongoing pregnancy (4).

For frozen embryo transfer (FET), the endometrium is frequently artificially prepared with estrogen and progesterone supplementation in order the match the endometrial stage during the critical implantation window. Women preparing for FET will often require additional estrogen supplementation, or other medication, if their endometrium is thin (3). Conventional treatments for inadequate endometrium including extended estrogen administration, Low-dose aspirin, vaginal sildenafil, pentoxifylline and tocopherol, have had spotty success at best              (5-   8 ). More recently, granulocyte colony stimulating factor has been suggested for the management of poor endometrial thickness (9, 10).

Many cytokines, identified as the product of inflammatory and immune cells, are expressed in human uterine tissue. These cytokines, appear to play a key role in varies endometrial functions, including the endometrial receptivity and early embryonic development granulocyte colony-stimulating factor (G-CSF) is a cytokine that produced primarily by hematopoietic cells. Recently G-CSF and G-CSF receptors have been also shown to be produced by endometrium, decidua and placenta, that suggesting that it may play a role in decidual and placental function Calhoun *et al* described the presences of G-CSF and its receptor in various human fetal tissues to (11, 12).

In 2011, Gleicher *et al* first used intrauterine G-CSF in four patients undergoing IVF with thin endometrium after standard endometrial preparation. All the patients successfully underwent ET and conceived. This demonstrates G-CSF as an novel remedy in patients with unresponsive, inadequate, and thin endometrium (9). Subsequently, the same author conducted a pilot cohort study of G-CSF in 21 patients. In their study endometrial thickness increased from 6.4-9.3 mm on average. They also suggests that G-CSF treatment may, in general, improve IVF pregnancy chances (10). This novel therapy demonstrated a hopeful result. The above-mentioned studies involved women with thin endometrium in a stimulated IVF program. 

More over G-CSF treatment is still a new remedy and the efficacy of G-CSF in improving endometrium, and possibly pregnancy rates, needs to be confirmed in randomized controlled trials. We planned this study to investigate whether endometrial perfusion with G-CSF is able to improve endometrial thickness and pregnancy outcomes in women with thin endometrium in FET cycles.

## Materials and methods

This interventional, non-randomized clinical trial was performed at the Yazd Research and Clinical Center for Infertility, Shahid Sadoughi University of Medical Sciences between September 2013 and March 2014. The study was approved by the Ethics Committee of the university. Informed written consent was obtained from all couples. 68 women with endometrial thickness below 7mm at 12^th^-13^th^ day of frozen-thawed embryo transfer cycle were allocated in this study. Patients less than 20 years, older than 40 years, those with BMI>30 Kg/m^2^, history of endocrine disorders, systemic diseases, severe endometriosis, repeated (≥3) implantation failure, repeated (≥3) abortion, congenital or acquired uterine anomaly, were excluded from the study.

Also third party reproduction cycles were excluded. The patients were allocated into two groups: G-CSF cotreated group and control group. Considering that G-CSF administration was an experimental treatment, based on the antecedent reports, the choice of the patients affects their following treatment. Both the patients and the physicians were aware of the allocated arm. The artificial endometrial preparation program was similar in two groups. 

All women received estradiol valerate tablet (2 mg, Aburaihan Co., Tehran, Iran) 6 mg daily and low-dose aspirin (80 mg, Pars Darou Co., Tehran, Iran) from the second day of the menstrual cycle. Ultrasonography was done from the 12^th^-13^th^ day of cycle. Endometrial thickness was measured at its thickest part in the longitudinal axis of the uterus. When the endometrial thickness was below 7 mm, the same ultra-sonographers measured it repeatedly for 2 times to confirm thin endometrium, and the average value of the 2 different measurements was recorded. 

The estradiol was increased to 8 mg in both groups. 34 patients received uterine infusion of 300 microgram recombinant human G-CSF (300 microgram, Zahravi Co., Tehran, Iran) by the use of IUI catheter, while the standard treatment was continued in the 34 patients who did not accept G-CSF treatment. After 2-3 days, the thickness of endometrium was measured again and if it was at least 7mm, the patient received 100mg intramuscular progesterone in oil (50 mg; Aburaihan Co., Tehran, Iran) for 3 days. Then 2 or 3, day-3 embryos were transferred. If not, the second injection was done in GCSF group and continued estradiol with 8 mg per day in control group. 

The cycle was cancelled in the patients with thin endometrium (endometrial thickness below 7mm) until 19^th^ cycle day ultimately. Estradiol, progesterone and aspirin were continued after the transfer and till the tenth week of gestational age if pregnancy occurred. Vitrification and warming was performed according to vitrolife instruction. Warmed embryos were transferred to a culture media and evaluated 1 day later. The criteria for embryo quality was consulted from the embryo morphology assessment according to Hill *et al* criteria, and cleavage-stage embryos scored as grade A, B,C and D. The grade D embryos were not transferred. 

The primary outcome was the expansion of endometrial thickness that was measured by serial vaginal sonography from the 12^th^-13^th^ day of transfer cycle till 19^th^ day ultimately. The secondary outcomes were chemical pregnancy that was determined by serum βhCG titer, 14 days after embryo transfer and clinical pregnancy that was determined by presence of gestational sac in vaginal sonography 28-30 days after embryo transfer.

**Statistical analysis**

Statistical analysis was performed using the Statistical Package for the Social Sciences (SPSS, version 16.0; SPSS Inc, Chicago, Illinois). Continuous data were presented as mean±SD and assessed by independent samples t-test between the groups. Enumeration data were compared by chi-square or fisher exact test. P<0.05 was considered significant.

## Results

A total of 68patients were included in this study. Among 68participants, 34women were treated with G-CSF and defined as G-CSF cotreated group, and 34 without G-CSF were defined control group. The baseline characteristics of the patients were summarized in (Table I). Basic characteristics including age, body mass index, type of infertility, number of prior embryo transfer, and cause of infertility were comparable between the patients who received G-CSF administration and those who refused. Cycle characteristic and outcomes of both groups showed in table II.

The proportion of thin endometrium in previous attempt for frozen-thawed embryo transfer was greater in G-CSF cotreated group (Table II). Mean of endometrial thickness (in the 12^th^-13^th^ cycle day) was similar in both groups (p=0.88.) (Table II). The endometrial thickness at the first day of progesterone administration, number and quality of transferred embryos, were similar in both groups. Thin endometrium was persistsntin5 patient with G-CSF treatment and 5 patients without G-CSF treatment.6 patients in G-CSF group needed 2 times infusion (18.18%). 2 patients were excluded from the final analysis due no suitable embryo for transfer, 1 patients receiving G-CSF treatment and 1 patients not. The cycle cancelation rate owing to thin endometrium was similar in G-CSF group (15.20%), followed by (15.20%) in the control group (p=1.00).

1 patient in G-CSF group developed ectopic pregnancy. Higher chemical (39.30% vs. 14.30%) and clinical pregnancy rates (32.10% vs. 12.00%) were observed in patients with adequate endometrial thickness after G-CSF treatment in comparison with the control group although were not statistical significant. It might be a result of the mechanism of GCSF effect in increasing LIF and decreasing CD16, CD 56 which is one of the most important factors in implantation. We were worried about the safety and side effects of G-CSF. We did not find any side effect at all.

**Table I:** Baseline characteristics in G-CSF cotreated and control groups

**Variables**		**G-CSF cotreated group (n= 34)**	**Control group (n=34)**	**p-value**
Age (years)		30.81 ± 4.60	28.57 ±5.16	0.06
BMI (kg/m^2^)		25.27 ± 1.48	24.93 ± 1.59	0.38
Type of infertility				
	Primary	27 (81.80%)	31 (93.90%)	0.12
	Secondary	6 (18.20%)	2. (6.10%)	
Etiology of infertility				
	Ovulatory	8 (24.20%)	5 (16.10%)	0.32
	Tubal	1 (3%)	1 (3.2%)	
	Male	11 (33.30%)	14 (45.20%)	
	Mixed	9 (27.30%)	11 (35.5%)	
	unexplained	4 (12.10%)	0 (0%)	

G-CSF: granulocyte colony-stimulating factor

BMI: body mass index

OHSS: ovarian hyperstimulation syndrome

IVF: in vitro fertilization.

Continuous data presented as mean±SD with p-values obtained from Independent- Samples T test; Enumeration data presented as N (%) with p-value obtained from Chi-Square or fisher exact tests. A p-value of<0.05 was considered statistically significant

**Table II:** Cycle characteristics and outcomes of FET cycles in G-CSF cotreated treated and control groups

**Variables **		**G-CSF cotreated group (n=34)**	**Control group (n= 34)**	**p-value**
No. of Transferred embryos		2.46 ± 0.63	2.39 ± 0.49	0.64
Embryo quality				
	A	20 (71.40%	17 (60.70%)	0.57
	B	8 (28.60%)	11 (39.30%)	
Cycle cancellation		5 (15.20%)	5 (15.20%)	1.00
Endometrial thickness, (mm) at				
	D1	5.63 ± 0.78	5.76 ± 0.86	0.88
	D2	7.91 ± 0.55	8.23 ± 0.82	0.10
Chemical pregnancy		11 (39.30%)	4 (14.30%)	0.68
Clinical pregnancy		9 (32.10%)	3(12%)	0.10

G-CSF: granulocyte colony-stimulating factor

FET: frozen embryo transfer

D1: endometrial thickness at the 12^th^-13^th^ day of frozen- thawed embryo transfer cycle

D2: endometrial thickness at the first day of progesterone injection

Transfer day^x^: The frozen- thawed cycle day which the embryos were transferred

Continuous data presented as mean±SD with p-values obtained from Independent-Samples T test; Enumerationl data presented as N (%) with p-value obtained from Chi-Square or fisher exact test. A p-value of<0.05 was considered statistically significant.

**Figure 1:**
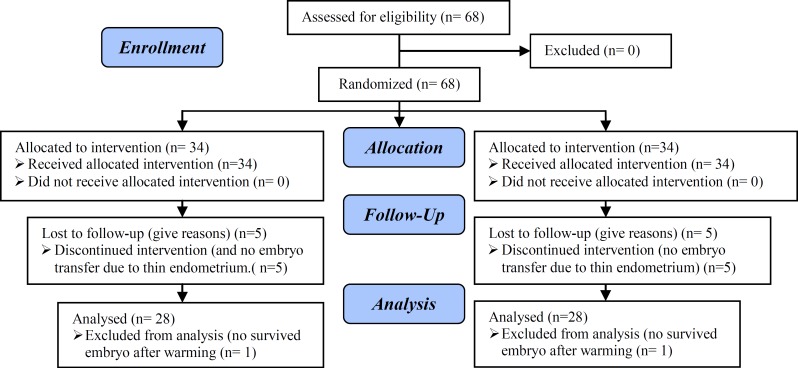
Consort flow chart

## Discussion

An adequate thickness of endometrium is essential for a successful pregnancy. In clinical practice, many researchers have indeed found the chances of pregnancy decreased when the endometrial thickness was 7 mm or even less (4). Inventive use of G-CSF in thin endometrium by Gleicher *et al* a rose our great interests (9, 10). In the current study, we used G-CSF in FET cycles of thin endometrium, in our study, the treatment protocol modalities of the G-CSF cotreated and control groups were no different. 

We found no difference in endometrial growth between 2 groups. The cycle cancelation rate due to unresponsive thin endometrium was similar in 2 groups too. The endometrial growth was not different within 2 groups. An clinical improvement was shown between controlled and G-CSF cotreated groups, with chemical (39.30% vs. 14.30%) and clinical pregnancy rates (32.10% vs. 12.00%) but due to small sample size these were not statistical significant. So endometrial Growth appeared to be unaffected by G-CSF treatment, but the outcome of pregnancy improved. Gleicher *et al* found a growth spurt in endometrial thickness within 48 hours of G-CSF administration during IVF cycle stimulation (9, 10). 

On the basis of our limited experiences in frozen-thawed embryo transfer cycle, we found no difference. Expansion of endometrial thickness in Gleicher *et al* study can be due to synergistic effects between G-CSF and sildenafil citrate, which all the patients in that study received before and during G-CSF administration. Li *et al* failed to demonstrate that G-CSF has the potential to improve embryo implantation and clinical pregnancy rate of the infertile women with thin endometrium in frozen-thawed embryo transfer cycles. They also found no marked difference in endometrial thickness before and after G-CSF infusion. This study was nonrandomized, retrospective and small sample size, and the women with G-CSF treatment seemed to have poorer endometrium than those without G-CSF treatment. Their administration of G-CSF was a relatively low dose (100 microgram) and only once (13).

Kim *et al* administered this cytokine in women with recurrent IVF failure due to poor endometrial development. Transvaginal endometrial perfusion with granulocyte colony-stimulating factor (G-CSF) enhanced endometrial development inpatients without synechea. Ongoing pregnancy and implantation rates were significantly higher. In patients with intrauterine synechea did not achieve an endometrial expansion (14). In clinical reproduction, G-CSF successfully has been applied as a treatment for implantation failure, repeated miscarriages and poor ovarian response (   15 -18). In these conditions, G-CSF has been administered by the subcutaneous rather than the intrauterine route. Which delivery method for the drug are superior remains to be determined. In addition, in these studies G-CSF was administered for much longer time periods, suggesting that more frequent administration may have superior benefits.

G-CSF, GM-CSF and M-CSF are, however, distinct: G-CSF facilitates stem cell and progenitor proliferation in neutrophilic granulocytes, while GM-CSF facilitates proliferation and differentiation in macrophages, granulocytes and eosinophils. Because of proliferative effects on fibroblasts, one, therefore, can hypothesize that GM-CSF may expand endometrial thickness more than G-CSF and M-CSF or GM-CSF may have even better effectiveness on endometrial thickness (10). Local GCSF significantly is decreasing CD16 & CD56 and it is also increases LIF so as a result the chances of getting pregnant are going to be improved; further studies are appreciated. We think that exogenous G-CSF infusion as chemical stimuli and intrauterine infusion as mechanical stimuli may induce secretion of endogenous cytokines and activated the endocrine-paracrine pathways that probably contributed to embryo implantation and pregnancy. 

Finally, we would like to point out the limitation of our study. This study was nonrandomized, and small sample size. Our study demonstrates that G-CSF has the potential to improve embryo implantation and clinical pregnancy rate of the infertile women with thin endometrium. This observation, however, has to be confirmed by larger prospective RCT. We would like to conduct further studies in terms of different dose and times for G-CSF administration to reveal the efficiency. 

## Conclusion

Our study fails to demonstrate that G-CSF has the potential to improve endometrial thickness but has the potential to improve chemical and clinical pregnancy rate of the infertile women with thin endometrium in frozen-thawed embryo transfer cycle. It might be a result of the mechanism of GCSF effect in increasing LIF and decreasing CD16, CD56 which is one of the most important factors in implantation.

## Footnote

***Registration ID in IRCT: IRCT201108116420N8***

***This article extracted from fellowship course thesis. (Mozhgan Sayadi)***
